# Predicting Physical Activity Intentions, Habits, and Action Plans in Finnish Parent–Child Dyads

**DOI:** 10.1111/sms.70028

**Published:** 2025-02-12

**Authors:** Daniel J. Phipps, Milla Saarinen, Weldon T. Green, Taru Lintunen, Keegan Knittle, Martin S. Hagger

**Affiliations:** ^1^ Faculty of Sport and Health Sciences University of Jyväskylä Jyväskylä Finland; ^2^ School of Applied Psychology Griffith University Mount Gravatt Australia; ^3^ Norwegian Research Center for Child and Youth Sport Norwegian School of Sport Sciences Oslo Norway; ^4^ Health Sciences Research Institute University of California—Merced Merced California USA; ^5^ Department of Psychological Science University of California—Merced Merced California USA

**Keywords:** autonomous motivation, habit, physical activity

## Abstract

Physical activity levels among early adolescents and their parents are insufficient for health benefits. Identifying modifiable determinants in parent–child dyads can inform future research and interventions. We tested a partial least squares path model based on the integrated behavior change model in insufficiently active Finnish parent–child dyads (*n* = 88), including measures of autonomous and controlled motivation, social cognition constructs (attitude, subjective norm, perceived behavioral control), intention, planning, and habits. Autonomous motivation predicted attitude in both samples, but only predicted subjective norms and perceived behavioral control in children. Attitude in turn predicted intention, planning, and habit, in the child sample, but only intention and planning in parents. Perceived behavioral control predicted intention and planning only in children, while subjective norm had minimal effects in either sample. Autonomous motivation and attitude consistently determined intention and planning for both parents and children, highlighting their importance in future research and interventions.

## 1 Introduction

Insufficient regular physical activity is associated with a myriad of maladaptive physical and psychological health consequences including increased risk of chronic disease [1, 2]. Yet, the majority of people in many developed countries are not sufficiently active for good health based on national guidelines. For example, only 37% of the Finnish population meets recommended activity levels [3]. While physical activity levels are insufficient across all segments of the population, two demographic groups that might be of particular interest are parents of young children and pre‐adolescent children aged 8–12 years old [4, 5]. That is, parents are significantly less active than adults without children [6], and only 47% of Finnish pre‐adolescent children meet physical activity recommendations, which may indicate declining levels of activity given that 67% of younger children meet recommendations [7]. As a consequence, these groups have been identified as priority targets for public health interventions aimed at promoting physical activity given evidence that childhood and adolescent inactivity tends to track into adulthood and later life [5].

One approach to physical activity promotion interventions may be to develop strategies targeting behavior change in groups or dyads, that is, pairs of people such as couples or parents and their children together, where both parties benefit from the intervention. Such approaches are considered attractive based on research suggesting that co‐action and mutual social support may be conducive to facilitating behavior change in health behavior contexts [8, 9]. However, in order to develop interventions comprising appropriate targeted strategies for dyadic behavior change, it is important to identify the determinants and processes underlying the targeted behavior in these groups. Accordingly, the current study was purposed to identify the determinants of physical activity habits, intentions, and plans in low‐active parent–child dyads and test a model comparing how the effects of model constructs differ between parents and children.

Theorists and researchers in the field of behavior change have advocated for the application of behavioral theories, particularly those from social psychology, to identify candidate determinants of health behavior [10]. This is based on the assumption that the psychological processes represented by theory‐based determinants are potentially modifiable and, as a consequence, may form viable targets for interventions purposed to change behavior [11, 12].

Eschewing an approach fixated on singular theories, researchers have developed multi‐theory integrated approaches to provide comprehensive descriptions of behavior, a prominent example of which is the integrated behavior change model [13]. The model draws its constructs and predictions from four commonly‐adopted social psychological perspectives that have been applied to predict and change physical activity behavior: the theory of planned behavior [14], self‐determination theory [15], dual phase models [16, 17], and dual process theory [18].

A central prediction of the integrated behavior change model is that effects of autonomous forms of motivation from self‐determination theory on behaviors like physical activity are mediated by an individuals' beliefs with respect to future performance of the behavior. Autonomous motivation reflects individuals' perceptions that their actions emanate from the self and are performed out of a sense of choice and volition and consistent with values and needs. In the model, beliefs are represented by constructs from the theory of planned behavior, a prototypical social cognition theory, specifically attitudes (beliefs that exercise will result in salient outcomes), subjective norms (beliefs that important others endorse their physical activity), and perceived behavioral control (beliefs in capacity to perform the behavior and to overcome barriers to its performance). The proposed mediated effect is based on the premise that individuals citing autonomous motives to be active have a greater likelihood of forming beliefs favoring performing the behavior in future, and these beliefs are the direct antecedent of intentions toward, and subsequent participation in, physical activity. Studies have supported the proposed mediation effect in numerous health behavior contexts [19, 20], including physical activity [13].

In addition, evidence suggests that autonomous motives, and the decision‐making process represented by beliefs and intention, leads not only to uptake but also persistence with behaviors [21, 22, 23]. This is consistent with the central self‐determination theory premise that holding autonomous motives as the impetus for action is perceived to emanate from the self and is not reliant on external reinforcements or contingencies. Accordingly, the increased likelihood that individuals citing autonomous reasons will consistently intend to and perform physical activity may also be conducive in habit formation given that consistent behavioral performance in the face of stable contexts or cues is critical for habit formation [24, 25]. This has been confirmed in research linking autonomous motivation and measures of habit [26]. In contrast, controlled forms of motivation from self‐determination theory that reflect perceptions that actions are performed at the behest of others or controlled by external events, are not likely to be related to beliefs that line up intentions to perform a behavior, and are not conducive the to formation of physical activity intentions and habits [27].

A further key prediction of the integrated behavior change model is that individuals will also furnish their intentions with plans to perform the behavior, consistent with dual phase models [16, 17] suggesting that augmenting intentions with detailed plans (e.g., stating when, where, and how the behavior might be performed) will lead to more efficient recall and enactment of the behavior [28]. Finally, the model also implies that individuals' beliefs may ultimately lead to habit formation. Habit represents non‐conscious behavioral responses to contextual cues developed through repeated behavioral performance coinciding with the cue over time [24, 29]. Researchers suggest that habits develop from behaviors that are initially controlled through the conscious, belief‐based processes represented by constructs from social cognition theory—a process which may be reflected in associations between belief‐based constructs and habits, often captured by experiences of the behavior as automatic, unthinking, and effortless. As before, those with autonomous motives toward a given behavior have a greater propensity to seek out and persist with that behavior, conditions conducive to habit formation as well as aligning their beliefs with the behavior [26, 30]. This is represented by the indirect effect of autonomous motivation on habit mediated by these sets of beliefs.

To date, studies applying variants of this model have provided some support for its validity in predicting health behaviors [19, 20], including physical activity [31, 32]. However, the majority of model tests have been in studies on older adolescent or adult samples [31], and it has yet to be applied in the context of dyads, such as parents and their children. An application in samples in this context may be of particular importance given evidence that the determinants of physical activity in children and adolescents may differ to those of their parents [33, 34, 35]. For example, children may cite more controlled rather than autonomous motives for physical activity if their activities tend to be supervised or restricted by their parents [36]. Their intention formation may also be highly related to their level of perceived behavioral control, reflecting their general dependence on parental assistance or approval to engage in many activities (e.g., being transported to sporting facilities, needing permission to go outside to play). Further, children, particularly pre‐adolescents, generally tend to express high motives for parental approval [37], which may manifest in effects of subjective norms on behavioral intentions [33]. Thus, while the predicted overall pattern of effects proposed in the integrated behavior change model is likely to hold across age groups, in keeping with its position as a generalized model of behavior, it is likely that the relative size of these effects may vary across populations, such as those from different developmental and age groups. Accordingly, identifying variation in the relative contribution of each model determinant on physical activity intentions and other key outcomes (e.g., planning and habit) across children and parents in dyads might assist in identifying appropriate determinants to be targeted in physical activity promotion interventions for each group or dyadically.

## 2 The Present Study

The current study aims to test hypothesized effects of the integrated behavior change model in a sample of low‐active parent child dyads and compare the effects across parents and children. We hypothesize that autonomous and controlled motivation will predict the social cognition constructs of attitude, subjective norms, and perceived behavioral control in both parents and children, but the effects of controlled motivation on the social cognition constructs will be larger in children than parents. Further, we expect that attitude, subjective norm, and perceived behavioral control will predict intentions, plans, and habits, but the effects of subjective norms and perceived behavioral control will be larger in children relative to their parents. Findings are expected to elucidate the theory‐based determinants of physical activity behavior within parent–child dyads, and to establish whether the relative importance of determinants differs between parents and children. Findings are also expected to contribute to an evidence base of potentially manipulable determinants of physical activity participation in parent–child dyads that could inform interventions aimed at promoting physical activity participation. Critically, the current research may provide important information on the extent to which these determinants vary across the parent and child groups within these dyads and signal points of similarity or difference in intervention content that might lead to optimal change in each dyad member.

## 3 Methods

### 3.1 Participants and Procedure

Low‐active Finnish parent–child dyads (*N* = 88) were recruited using social media advertisements and a panel company as part of the [38]. Dyads were eligible to participate if the child was aged between 8 and 12 years old, both the parent and child reported not being sufficiently active (i.e., parents and children reporting not meeting guideline levels of 30 min of moderate‐to‐vigorous physical activity per day on 5 or more days a week for adults and 60 min of physical activity per day for children, respectively), and, neither the parent or child had any condition that might prohibit them from being physically active. After providing informed consent, participants were contacted by email with links to separate surveys for the parent and child. Parents were instructed that they may help their child in understanding the survey content but should not attempt to influence the responses of their children. Complete data were available for analysis from 88 parents (3 fathers, 84 mothers, 1 non‐binary) and 75 children (44 boys, 31 girls).

### 3.2 Measures

Measures were adapted from previous research in the Finnish context [39]. Measures were initially piloted for readability in a group of Finnish children (*N* = 4) aged between 8 and 12 years, and subsequently refined for readability based on feedback received. Full measures and scales are available in Table S1.

#### 3.2.1 Autonomous and Controlled Motivation

Autonomous and controlled motivation were each assessed using four items with the common stem: “I (would) do physical activity during my free time…” (e.g., “…Because I enjoy doing physical activity”, “…Because I will feel guilty if I do not do physical activity”). Responses were provided on five‐point Likert scales (1 = strongly disagree to 5 = strongly agree) [40].

#### 3.2.2 Attitude

Attitude toward leisure time physical activity was assessed using three semantic differential items with the common stem “Participating in physical activity during my leisure time over the next 5 weeks is…”, with responses provided on five‐point bipolar scales (e.g., 1 = bad to 5 = good) [41].

#### 3.2.3 Subjective Norm

Subjective norm was assessed using two items (e.g., “Most people who are important to me think I should be physically active during my leisure time over the next 5 weeks.”), with responses provided on five‐point Likert scales (1 = strongly disagree to 5 = strongly agree) [41].

#### 3.2.4 Perceived Behavioral Control

Perceived behavioral control was assessed using two items (e.g., “How much control do you have over being physically active in your leisure time over the next 5 weeks?”), with responses provided on five‐point scales (1 = very little control to 5 = complete control) [41].

#### 3.2.5 Intention

Intention to be physically active was assessed using two items (e.g., “I intend to be physically active during my leisure time over the next 5 weeks.”), with responses provided on five‐point Likert scales (1 = strongly disagree to 5 = strongly agree) [41].

#### 3.2.6 Planning

Planning was assessed using a single item, “I have a clear plan of when, where and how I will be physically active during my leisure time over the next 5 weeks” with responses provided on a five‐point Likert scale (1 = strongly disagree to 5 = strongly agree).

#### 3.2.7 Habit

Habit was assessed using the behavioral automaticity scale [42], a variation of the self‐reported habit index [43]. The scale consisted of four items (e.g., “Physical activity is something I do automatically”) with responses provided on five‐point Likert scales (1 = strongly disagree to 5 = strongly agree).

### 3.3 Data Analysis

Data were analyzed using linear partial least squares structural equation modeling using the WarpPLS version 8.0 analysis software [44]. We used composite (averaged) scaled constructs to indicate each latent variable in the model. Standard errors were calculated using the ‘Stable 3’ method [45]. Model fit and quality were assessed using the Tenenhaus goodness of fit index (acceptable if > 0.25, assuming medium effect sizes), AVIF (acceptable if < 5), SSR (acceptable if > 0.70), RSCR (acceptable if > 0.70), and SPR (acceptable if > 0.70) [46]. We used multigroup analysis to estimate differences in model effects across the parent and child groups implemented using the Satterthwaite method. Item weights and loadings were also compared between parents and children using the Satterthwaite method to ensure observed model differences may not be attributed to parents and children interpreting and responding to items differently, analogous to measurement invariance testing in maximum likelihood based structural equation models. Following PLS recommendations [47], a minimum sample of 59 was required to achieve a power of 0.80, assuming a medium sized effect (*R* ^2^ = 0.25) and an alpha of 0.05.

## 4 Results

**FIGURE 1:**
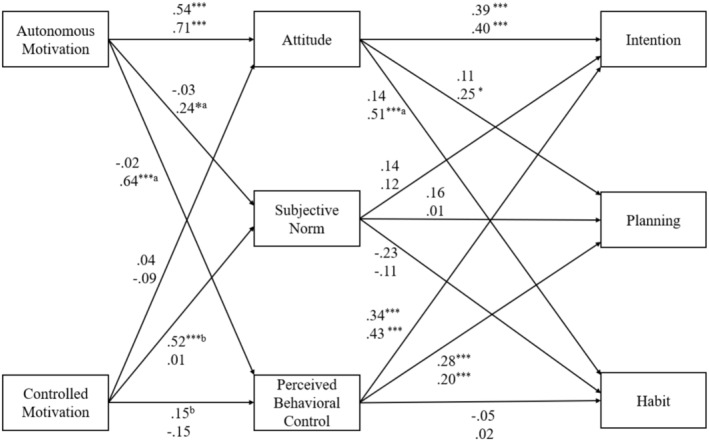
Results of a structural equation model of the integrated behavior change model predicting intention, planning, and habit in parent child Dyads. Effect sizes shown are standardized parameter estimates, with estimates from the model estimated in the parent sample are printed on the upper line and estimates from the model estimated in the child sample printed on the lower line. ^a^Standardized parameter estimate is larger in the child model compared to the parent model. ^b^Standardized parameter estimate is larger in the parent model compared to the child model. *p < 0.05, **p < 0.01, ***p < 0.001.

Descriptive statistics, bivariate correlations, and reliability statistics are available in Table 1. The model showed good fit with the data (Parent model: GoF = 0.452, AVIF = 1.08, SSR = 0.80, RSCR = 1.00, SPR = 1.00; Child model: GoF = 0.589, AVIF = 1.31, SSR = 0.86, RSCR = 0.99, SPR = 0.93), with no differences in indicator weights or loadings between parents and children (see Table S1). Parameter estimates for the model are presented in Table 2, and a diagram of the tested model is presented in Figure 1.^1^

**TABLE 1:** Bivariate correlations, descriptive statistics, and reliability coefficients for study constructs.

Construct	1	2	3	4	5	6	7	8	*M*	SD	*α*
1. Autonomous motivation	—	−0.06	0.71***	0.24*	0.65***	0.67***	0.43***	0.42***	3.80	0.78	0.83
2. Controlled motivation	0.04	—	−0.13	−0.01	−0.18	−0.13	−0.07	0.04	2.47	0.81	0.75
3. Attitude	0.54***	0.07	—	0.25*	0.64***	0.71***	0.38***	0.50***	4.04	0.81	0.74
4. Subjective norm	−0.01	0.52***	0.23*	—	0.23	0.32**	0.12	0.02	4.36	0.57	0.55
5. PBC	−0.01	0.15	0.27*	0.30**	—	0.72***	0.37**	0.33**	3.96	0.80	0.51
6. Intention	0.45***	0.14	0.51***	0.33**	0.49***	—	0.53***	0.36**	3.79	0.92	0.75
7. Planning	0.26*	0.16	0.22*	0.27*	0.35***	0.48***	—	0.35**	2.83	1.13	—
8. Habit	0.47***	−0.11	0.07	−0.21*	−0.08	0.13	0.16	—	3.24	0.94	0.88
*M*	3.76	2.87	4.17	3.94	3.82	4.16	2.63	2.29			
SD	0.73	0.88	0.78	0.73	0.83	0.69	1.19	0.87			
*α*	0.76	0.75	0.74	0.60	0.67	0.72	—	0.90			

*Note:* Statistics above the principal diagonal refer to the child sample, while statistics below the principal diagonal refer to the parent sample.

* *p* < 0.05.

** *p* < 0.01.

*** *p* < 0.001.

**TABLE 2:** Parameter estimates for the model predicting intention, planning, and habit in parent–child Dyads.

	Parents		Children		*p* Diff.
	*β*	*p*	*β*	*p*	
Autonomous motivation→Attitude	0.54	< 0.001	0.71	< 0.001	0.198
Autonomous motivation→Subjective norm	−0.03	0.386	0.24	0.014	0.072
Autonomous motivation→PBC	−0.02	0.419	0.64	< 0.001	< 0.001
Controlled motivation→Attitude	0.04	0.337	−0.09	0.226	0.401
Controlled motivation→Subjective norm	0.52	< 0.001	0.01	0.481	< 0.001
Controlled motivation→PBC	0.15	0.069	−0.15	0.095	0.047
Attitude→Intention	0.39	< 0.001	0.40	< 0.001	0.895
Attitude→Planning	0.11	0.154	0.25	0.012	0.347
Attitude→Habit	0.14	0.096	0.51	< 0.001	0.008
Subjective norm→Intention	0.14	0.094	0.12	0.134	0.936
Subjective norm→Planning	0.16	0.060	0.01	0.457	0.337
Subjective norm→Habit	−0.23	0.013	−0.11	0.160	0.445
PBC → Intention	0.34	< 0.001	0.43	< 0.001	0.540
PBC → Planning	0.28	0.003	0.20	0.031	0.632
PBC → Habit	−0.05	0.310	0.02	0.427	0.637
Indirect effects					
Autonomous motivation→Attitude→Intention	0.21	0.002	0.29	< 0.001	0.440
Autonomous motivation→Subjective norm→Intention	0.00	0.478	0.03	0.357	0.786
Autonomous motivation→PBC → Intention	−0.01	0.461	0.28	< 0.001	0.007
Autonomous motivation→Intention^a^	0.20	0.027	0.59	< 0.001	0.006
Autonomous motivation→Attitude→Planning	0.06	0.221	0.17	0.014	0.305
Autonomous motivation→Subjective norm→Planning	0.00	0.474	0.00	0.485	0.999
Autonomous motivation→PBC → Planning	−0.01	0.469	0.13	0.049	0.198
Autonomous motivation→Planning^a^	0.05	0.330	0.31	0.002	0.082
Autonomous motivation→Attitude→Habit	0.07	0.163	0.36	< 0.001	0.006
Autonomous motivation→Subjective norm→Habit	0.01	0.463	−0.03	0.371	0.718
Autonomous motivation→PBC → Habit	0.00	0.494	0.01	0.434	0.928
Autonomous motivation→Habit^a^	0.08	0.220	0.35	< 0.001	0.067
Controlled motivation→Attitude→Intention	0.02	0.410	−0.03	0.336	0.651
Controlled motivation→Subjective norm→Intention	0.07	0.169	0.00	0.500	0.527
Controlled motivation→PBC → Intention	0.05	0.241	−0.06	0.218	0.314
Controlled motivation→Intention^a^	0.14	0.087	−0.10	0.196	0.115
Controlled motivation→Attitude→ Planning	0.01	0.475	−0.02	0.399	0.786
Controlled motivation→Subjective norm→ Planning	0.08	0.130	0.00	0.497	0.470
Controlled motivation→PBC → Planning	0.04	0.288	−0.03	0.356	0.524
Controlled motivation→Planning^a^	0.13	0.372	−0.05	0.328	0.243
Controlled motivation→Attitude→ Habit	0.01	0.468	−0.04	0.295	0.651
Controlled motivation→Subjective norm→ Habit	−0.12	0.054	0.00	0.497	0.276
Controlled motivation→PBC → Habit	−0.01	0.458	0.00	0.485	0.928
Controlled motivation→Habit^a^	−0.12	0.498	−0.05	0.340	0.649

Abbreviations: PBC, Perceived behavioral control; *p* Diff., *p* values for Satterthwaite comparisons between parameter estimates in parents and children.

^a^ Coefficient is the sum of indirect effects of autonomous/controlled motivation on dependent variable mediated by all three social cognition constructs, attitude, subjective norms and perceived behavioral control.

Focusing first on the model estimated in the parent sample, the model predicted 41%, 16%, and 6% of the variance in intention, planning, and habit, respectively. We found non‐zero positive direct effects of autonomous motivation on attitude, controlled motivation on subjective norm, attitude and perceived behavioral control on intention, and perceived behavioral control on planning. We also found a non‐zero direct effect of subjective norm on habit, but, contrary to hypotheses, the effect was in a negative direction. Finally, we found a non‐zero positive indirect effect of autonomous motivation on intentions mediated by attitude.

In the child sample, the model explained 64%, 17%, and 26% of the variance in intention, planning, and habit, respectively. We observed non‐zero, positive direct effects of autonomous motivation on attitude, subjective norm, and perceived behavioral control, attitude and perceived behavioral control on planning, and attitude on habit. Finally, we observed non‐zero indirect effects of autonomous motivation on intentions and planning mediated by attitude and perceived behavioral control, and on habit mediated by attitude alone.

Multi‐group comparisons revealed that the effects of autonomous motivation on attitude and perceived behavioral control and of attitude on habit were larger in the model estimated in the child sample. Further, the indirect effects of autonomous motivation on intention, mediated by perceived behavioral control, and of autonomous motivation on planning, mediated by attitude, were also larger in the model estimated in children. By contrast, the effect of controlled motivation on subjective norm was larger in the model estimated in the parent sample.

## 5 Discussion

We tested an integrated behavior change model specifying theory‐based determinants of physical activity intentions, planning, and habit in a sample of insufficiently active parent–child dyads. The determinants represented key motivational, social cognition, and non‐conscious processes involved in physical activity participation. We also compared the size of the effects of these determinants on intentions, planning, and habit across parents and children.

Focusing first on the effects of motivational constructs from self‐determination theory on the social cognition constructs from the theory of planned behavior. We expected to observe effects of autonomous motivation on attitude, subjective norms, and perceived behavioral control [13, 31], but expected effects for controlled motivation to be trivial by comparison. Findings corroborated this expected pattern in the child sample—autonomous motivation was related to each social cognition construct. By contrast, we observed an effect of autonomous motivation on attitude alone in the parent sample, and also observed an effect of controlled motivation on subjective norms. The latter effect contrasts with theory and prior evidence indicating that autonomous motivation should be the most salient predictor of these belief based determinants of physical activity [31, 48, 49]. To speculate, the greater salience of controlled motivation in low‐active parents likely reflects the role of obligation as a determining factor among parents specific to this group, which is consistent with research indicating that guilt around engaging or not engaging in physical activity as a key factor in decisions to exercise in parents, especially those who are not particularly active [50].

Focusing on the effects of social cognition constructs from the theory of planned behavior and planning from dual phase models [14, 17], results were consistent with theory insofar as we observed effects of attitude and perceived behavioral control on intention in both samples, and effects of attitude and perceived behavioral control on planning in the child sample, but only of perceived behavioral control in the parent sample. Contrary to predictions, subjective norm predicted neither intention nor planning in either sample [14, 51]. Overall, this pattern of effects is somewhat consistent with those observed elsewhere that has identified attitude and perceived behavioral control as key social cognition determinants of physical activity with smaller, but not trivial, effects for subjective norms [33]. Researchers have also indicated that the relative effects of these constructs on intention will likely vary in size, rather than pattern, according to salient moderators [33]. In the current research, we had anticipated larger effects for subjective norms on intention among children in the current study because we expected parental approval would be a key consideration for exercise in children [37]. However, given the trivial effect size and lack of difference we observed in subjective norm effects across the models estimated in the parent and child samples, it may be a valuable avenue for future research to investigate whether or not these effects are also corroborated in other low‐active parent–child dyads. It may also be useful to examine a broader gamete of constructs reflecting normative influence, given the suggestion that subjective norms is a rather narrow construct and may not capture other components of normative influence such as social support or descriptive and moral norms [52, 53].

We also expected that attitude, subjective norms, and perceived behavioral control would be associated with habit, as favorable beliefs around physical activity should engender regular behavioral engagement, and thus be conducive to habit formation, given consistent performance in stable contexts is a recognized prerequisite to habit formation [21, 25]. We found that attitudes alone were related to habits, consistent with the view that beliefs in the utility of behaviors in achieving salient outcomes will increase the likelihood of consistent behavioral performance over time [54, 55]. However, this effect was larger in children compared to parents, were the effect fell short of statistical significance. This aligns with prior findings indicating that outcomes such as enjoyment are more salient for children and more likely to drive behavior [56], which may contrast with parents who may place greater emphasis on social pressures and self‐efficacy beliefs [50]. However, these interpretations are speculative and require further corroboration focusing on the specific beliefs that underpin attitudes and other social cognition constructs and setting them as determinants of physical activity habits in parent and child samples.

A key prediction of the integrated behavior change model is that effects of autonomous motivation on intentions, planning, and habits should be mediated by individuals' beliefs with respect to future behavioral performance here represented by the social cognition constructs from theory of planned behavior [31]. Findings provided broad support for this contention—we found indirect effects of autonomous motivation on intention in both the parent and child samples, and on planning and habit in the child, but not the parent, sample. Attitude was the common mediator in all cases. Consistent with a core integrated behavior change model prediction and prior research [13], these findings indicate that those citing self‐endorsed reasons for physical activity are more likely to form beliefs that line up future performance of the behavior and increase the likelihood of intentions and plan formation, and, with sustained action in stable contexts over time, habits.

## 6 Implications, Limitations, and Future Directions

The current study provides an important test of the integrated behavior change model in parent–child dyads that has implications for the development of theory on determinants of physical activity in these dyads and may provide initial evidence to inform behavior change strategies. Our findings indicate prominent roles for autonomous motivation and attitude as determinants of physical activity intentions, planning, and habit in both parents and children and the associated theory‐based processes involved. Specifically, our findings suggest that future models and research on determinants should consider these constructs as priority and seek to include them as standard in prediction models to further develop an evidence base of the determinants of dyadic physical activity. Findings may also signal the potential for these determinants as potential intervention targets, and the possibility that techniques targeting change in them (e.g., autonomy supportive parenting technique or messaging promoting salient the outcomes of physical activity), may have utility in promoting physical activity in both parents and children. However, making definitive recommendations based on these findings would be premature, and more research that replicates these findings and extends them so as to secure better inferences is warranted before such conclusions could be drawn. It is also important to note that the observed differences in the model effects across the parent and child samples make it plausible that it may be more important to target different constructs in parents and children. However, again drawing a definitive conclusion for these recommendations based on these findings would be premature and further corroboration would be needed before doing so.

While the current study has inherent value in identifying potential determinants of physical activity intentions, planning, and habits in parent–child dyads, its findings should be considered in light of several key limitations. First, when comparing model effects across parents and children, it is important to consider that it is plausible parents and children may read and interpret self‐report items differently, or draw from different levels of experience when reporting their beliefs and expectations with respect to physical activity behaviors. A lack of statistically significant differences in item weights between parents and children provides some evidence to suggest that the impact of such differences on results were minimal. Nevertheless, this is still an issue of concern inherent to dyadic research—members of dyads may react differently to measures which may introduce bias when making comparisons between members. For example, when considering subjective norm items about the influence of important others on physical activity, there is evidence that parents might interpret such items mainly in consideration to partners [50], while children may consider their parents, peers, or a combination thereof depending on their age and the specifics of any given activity [57, 58]. Such differences are an important consideration for future research, particularly if attempting to translate model findings into practical behavior change strategies.

Second, the current study focused on intentions, plans, and habits with respect to being active, rather than physical activity behavior itself. While intentions, plans, and habits have all been associated with prospectively measured physical activity behavior in prior research [33, 59], relations between these constructs and behavior tend to be modest in size [60]. Thus, future research is required to assess the predictive validity of the model in explaining variance in prospective, observational measures of physical activity behavior. Further, the current research adopted a cross‐sectional design precluding directional or causal assertions in the model effects tested [61]. So, we been cautious in not drawing such inferences and stress that direction in model effects is based solely on prior evidence and theory not the data [31]. Future research should seek to adopt alternative designs including some forms of longitudinal design (e.g., cross‐lagged panel design) and experimental methods to corroborate these effects and permit directional and causal inferences. For example, studies adopting randomized designs could examine whether manipulations or techniques targeting change in key model constructs (e.g., autonomous motivation) lead to changes in outcomes of interest (e.g., social cognition constructs or intentions). Further, the current study made use of a relatively modest sample, comprised predominantly of female parents and all drawn from inactive members of the Finnish population. As before, we look to future studies that aim to corroborate these findings by replicating them in larger samples and dyads drawn from other populations and national groups.

Finally, it is important to note that reported model effects were small‐to‐medium in size and the model constructs only accounted for a modest proportion of the variance in intentions, planning, and habits. Such a finding is not surprising given similar sized effects and variance estimates have been noted in previous research applying the model, and other similar models of motivation and social cognition [33]. While the tested model included constructs derived from several theoretical frameworks, other unmeasured constructs likely accounted for the unexplained variance in physical activity intentions or represent additional processes that influence the translation of motives and beliefs into physical activity intentions and outcomes. Such constructs may include those that represent the influence, for example, of societal and family dynamics [62], psychological needs [15, 27], or affective or impulsive processes [32, 63]. Future research may seek to extend on the current model, integrating additional perspectives that may provide a more comprehensive description of the determinants of physical activity in parents and children.

## 7 Conclusions

The current study presents a novel test of an integrated behavior change model to identify determinants of physical activity intentions, planning, and habits in low‐active parent–child dyads. Findings supported key predictions of the proposed model in parent and child samples, including autonomous forms of motivation and attitude as key determinants of physical activity intention, planning, and habit. We observed some differences in the relative contribution of model constructs in the model in parents and children, such as larger effects for autonomous motivation on attitude for children relative to parents. Findings contribute to evidence of theory‐based determinants of physical activity intentions, planning, and habit and may indicate the potential for future research corroborating these findings in dyads in larger samples and from other national groups and may serve as a starting point for considering the dyadic intervention strategies that may have utility in promoting physical activity intentions, planning, and habits in parents and children through intervention.

## 8 Perspective

The current findings reaffirm support for the use of the integrated behavior change model as a tool for understanding physical activity behavior in the context of inactive parents and children. In terms of practical implications, our findings indicate potentially important roles for autonomous motivation and attitude as determinants of physical activity intentions, planning, and habit in both parents and children. Thus, in terms of physical activity behavior change, findings may also signal the potential value for these determinants as intervention targets, and the possibility that techniques targeting change in them (e.g., autonomy supportive parenting technique or messaging promoting salient the outcomes of physical activity), may have utility in promoting physical activity in both parents and children. Collectively, these findings indicate that the determinants of physical activity in parents and children are similar in many ways, but have some differences which may be worthy of consideration when attempting to encourage physical activity at a family level.

## Ethics Statement

All procedures were approved by the University of Jyväskylä Human Sciences Ethics Committee.

## Conflicts of Interest

The authors declare no conflicts of interest.

## Supporting information


Table S1.

## Data Availability

The data that support the findings of this study are openly available in Open Science Framework at https://osf.io/5py3a/, reference number 5py3a.

## Endnotes

^1^
For completeness, we also offer comparisons of model constructs between parents and children in Appendix B, online supporting information.
