# Supplementary material for: Predicting Physical Activity Intentions, Habits, and Action Plans in Finnish Parent–Child Dyads
Source: Scand J Med Sci Sports. 2025 Feb 12;35(2):e70028. doi: 10.1111/sms.70028 (PMC11822087; doi:10.1111/sms.70028)
Supplement: Supplementary file 1 — Table S1. [file SMS-35-e70028-s001.docx]

# Appendix A: Survey Materials

## Supplementary Table 1

| Construct | Item | Response Options | *p* loadings | *p* weights |
| --- | --- | --- | --- | --- |
| Autonomous Motivation | I (would) do physical activity during my free time… |  |  |  |
|  | ...Because I enjoy doing physical activity | [1] Strongly Disagree to [5] Completely Agree | .414 | .578 |
|  | ...Because I value the benefits of physical activity | [1] Strongly Disagree to [5] Completely Agree | .081 | .728 |
|  | ...Because it is fun | [1] Strongly Disagree to [5] Completely Agree | .701 | .867 |
|  | ...Because it’s important to me to do physical activities | [1] Strongly Disagree to [5] Completely Agree | .705 | .891 |
| Controlled Motivation | I (would) do physical activity during my free time… |  |  |  |
|  | ...Because I feel bad about myself when if don’t do physical activity | [1] Strongly Disagree to [5] Completely Agree | .419 | .746 |
|  | ...Because other people I know will not be pleased with me if I do not do physical activity | [1] Strongly Disagree to [5] Completely Agree | .761 | .928 |
|  | …Because I will feel guilty if I do not do physical activity | [1] Strongly Disagree to [5] Completely Agree | .755 | .924 |
|  | ...Because I feel under pressure from people I know to do physical activity | [1] Strongly Disagree to [5] Completely Agree | .794 | .939 |
| Attitude | Participating in physical activity during my leisure time over the next 5 weeks is… | [1] Unenjoyable to [5] Enjoyable | .637 | .825 |
|  |  | [1] Bad to [5] Good | .845 | .921 |
|  |  | [1] Useless to [5] Useful | .494 | .770 |
| Subjective Norms | Most people who are important to me think I should be physically active during my leisure time over the next 5 weeks. | [1] Strongly Disagree to [5] Completely Agree | .892 | .929 |
|  | Most people important to me put pressure on me to be physically active during my leisure time over the next 5 weeks. | [1] Strongly Disagree to [5] Completely Agree | .892 | .929 |
| Perceived Behavioral Control | How much control do you have over being physically active in your leisure time over the next 5 weeks? | [1] Very little control to [5] Complete Control | .526 | .690 |
|  | I am confident I could be physically active during my leisure time over the next 5 weeks. | [1] Strongly Disagree to [5] Completely Agree | .526 | .690 |
| Intention | I intend to be physically active during my leisure time over the next 5 weeks. | [1] Strongly Disagree to [5] Completely Agree | .852 | .914 |
|  | I want to be physically active during my leisure time over the next 5 weeks. | [1] Strongly Disagree to [5] Completely Agree | .852 | .914 |
| Planning | I have a clear plan of when, where and how I will be physically active during my leisure time over the next 5 weeks. | [1] Strongly Disagree to [5] Completely Agree | - | - |
| Habit | Physical activity is something I do automatically. | [1] Strongly Disagree to [5] Completely Agree | .926 | .897 |
|  | Physical activity is something I do without having to consciously remember. | [1] Strongly Disagree to [5] Completely Agree | .871 | .953 |
|  | Physical activity is something I do without thinking. | [1] Strongly Disagree to [5] Completely Agree | .688 | .996 |
|  | Physical activity is something I start doing before I realize I’m doing it. | [1] Strongly Disagree to [5] Completely Agree | .786 | .982 |
| *Note*. *p* loadings = the *p* value of the difference in items loadings between parents and children compared using the Satterthwaite method. *p* weights *=* the *p* value of the difference in items weights between parents and children compared using the Satterthwaite method. | | | | |

# Appendix B: Supplementary Analyses

## Supplementary Table 2

*Differences in integrated behavior change model construct means between parents and children*

|  | Parents | |  | Children | |  | Differences | | |  |  | Bivariate Correlation | |
| --- | --- | --- | --- | --- | --- | --- | --- | --- | --- | --- | --- | --- | --- |
|  | *M* | *SD* |  | *M* | *SD* |  | *t* | *p* | *d* |  |  | *r* | *p* |
| Autonomous Motivation | 3.80 | 0.78 |  | 3.76 | 0.73 |  | -0.35 | .730 | -0.04 |  |  | .103 | .377 |
| Controlled Motivation | 2.47 | 0.81 |  | 2.87 | 0.88 |  | 3.71 | < .001 | 0.43 |  |  | .063 | .593 |
| Attitude | 4.04 | 0.81 |  | 4.17 | 0.78 |  | 1.65 | .103 | 0.19 |  |  | .007 | .955 |
| Subjective Norm | 4.36 | 0.57 |  | 3.94 | 0.73 |  | -3.85 | <.001 | 0.44 |  |  | .315 | .006 |
| Perceived Behavioral Control | 3.96 | 0.80 |  | 3.82 | 0.83 |  | -0.40 | .693 | -.05 |  |  | .176 | .131 |
| Intention | 3.79 | 0.92 |  | 4.16 | 0.69 |  | 3.48 | < .001 | 0.40 |  |  | .074 | .530 |
| Planning | 2.83 | 1.13 |  | 2.63 | 1.19 |  | -1.03 | .307 | -0.12 |  |  | .348 | .002 |
| Habit | 3.24 | 0.94 |  | 3.76 | 0.73 |  | -8.02 | < .001 | -0.93 |  |  | .323 | .005 |
